# The relationship between spousal empowerment, quality of life, and subjective wellbeing among disabled elderly

**DOI:** 10.3389/fpsyg.2025.1606373

**Published:** 2025-07-08

**Authors:** Fei Ye, Yuanrong Wu, Chao Pan, Zifen An, Yanzhen Zhai, Dan Wang, Liping Yu, Yanni Zhu

**Affiliations:** ^1^The Fifth Affiliated Hospital, Southern Medical University, Guangzhou, China; ^2^School of Nursing and Health, Nanfang College Guangzhou, Guangzhou, China; ^3^Zhongnan Hospital of Wuhan University, Wuhan, China; ^4^Wuhan University School of Nursing, Wuhan, China

**Keywords:** disabled elderly, quality of life, wellbeing, spousal, empowerment

## Abstract

**Background:**

As rapidly ages, the number of disabled elderly is increasing, leading to lower quality of life and greater psychological stress.

**Aim:**

To explore the relationship between spousal empowerment, quality of life, and subjective wellbeing (SWB) among disabled elderly, providing insights and practical guidance for enhancing SWB in this demographic.

**Method:**

A convenience sampling approach was employed to select 332 disabled elderly and their spouses. Research tools included a demographic survey, the Barthel Index (BI), the World Health Organization Quality of Life Assessment - Older Adults Version (WHOQOL-OLD), the Memorial University of Newfoundland Scale of Happiness (MUNSH), and the Main Caregivers’ Empowerment Measurement (MCEM). Statistical analysis was performed using SPSS and AMOS.

**Result:**

The mean scores for the quality of life, SWB, and spousal empowerment were 72.07 ± 19.79, 24.13 ± 8.98, and 140.92 ± 29.13. Multiple linear regression analysis identified spousal empowerment (*β* = 0.258, *p* < 0.001) and the duration of disability (*β* = −0.142, *p* = 0.032) as significant predictors of SWB. The results of testing the mediating role of spousal empowerment using the structural equation model show that quality of life directly predicted SWB with a path coefficient of 0.208 (95% CI: 0.065, 0.289). Spousal empowerment partially mediated the relationship between quality of life and SWB, with a mediation effect of 0.067 (95% CI: 0.026, 0.098).

**Conclusion:**

Both quality of life and spousal empowerment can positively influence the SWB of disabled elderly. Additionally, spousal empowerment partially mediates the relationship between quality of life and SWB.

## 1 Introduction

With the global trend of aging, China has rapidly transitioned into an aging society, marked by a significant increase in the proportion of geriatric. By 2023, elderly individuals had reached 296 million, accounting for 21.1% of the total population, a 5.4% increase from 2010 (National Bureau of Statistics, 2024). This trend is expected to continue until 2050, highlighting the large-scale, rapid growth of aging in China. The risk of chronic diseases and physical impairments escalates with aging, leading to a continuous rise in the number of elderly individuals with disabilities (World Health Organization, 2015). In 2016, the number of disabled and semi-disabled elderly individuals in China surpassed 40.63 million (Wu, 2015). It is projected to increase to 61.68 million by 2030, with a sharp rise to 97.5 million by 2050 (Zhao and Zhang, 2022). This indicates that the intensification of aging will inevitably lead to a continued increase in disabled elderly.

In the 2016 Global Report on Aging and Health (Beard et al., 2016), the World Health Organization (WHO) highlighted the primary objective of healthy aging, which is to preserve the functional integrity of elderly individuals, promote social participation, and improve quality of life, thereby extending healthy life expectancy. This has become a global consensus and a central policy direction for China in addressing the challenges of its aging population. The age threshold aligns with China’s official definition of “elderly” in the Law on the Protection of the Rights and Interests of the Elderly (2018 revision) and the Healthy China 2030 initiative, which set 60 years as the standard for elderly care policies. In low- and middle-income countries (LMICs) including China, the WHO recommends 60 + years as the benchmark for aging studies (World Health Organization, 2024), as this group faces higher disability risks and care dependency. Elderly individuals with disabilities, due to their physical vulnerability, generally suffer from low quality of life and significant psychological stress (Ahmad et al., 2020). Many countries have implemented a range of interventions, such as physical activity programs (Groessl et al., 2019), home-based smart management systems (Xue et al., 2014), and multi-level intervention models based on health ecology theory (Chang et al., 2018), aimed at improving the quality of life of disabled elderly. However, there remains a notable gap in the theoretical framework that guides the development of interventions from a positive psychology perspective.

Subjective wellbeing (SWB) is a critical indicator of quality of life in elderly individuals and is closely linked to their health status. High levels of SWB not only represent enhanced quality of life but also play a vital role in disease prevention and mortality reduction (Sun, 2023). Studies have shown a reduced risk of disability and prolonged life expectancy among elderly individuals with higher levels of SWB (Zaninotto and Steptoe, 2019). Moreover, a significant negative correlation exists between the degree of disability and SWB, indicating that the more severe the disability, the lower the level of wellbeing (Minagawa and Saito, 2023). In China, factors such as material security, social connections, and mental health significantly impact the wellbeing of disabled elderly (Ding, 2017). Positive psychological traits, such as resilience and optimism, help disabled elderly manage challenges in their daily lives and ameliorate interpersonal relationships, thereby enhancing their SWB.

Currently, the care system for elderly individuals with disabilities in China is primarily characterized by “home as the foundation, community as the support, and institutions as the supplement,” which encompasses both formal and informal care types. Although healthy aging and care services are aggressively promoted by policies, the socialized care system remains underdeveloped (He et al., 2023). Moreover, the professional care market has issues such as insufficient supply and high costs. Consequently, family-based care continues to dominate the care model for disabled elderly. In this context, spouses, as the primary caregivers, bear a significant responsibility. They not only provide physical care and emotional support but also facilitate the dissemination of health knowledge and encourage the self-health management potential of their partners (Lin et al., 2022). Empowerment, which enhances caregivers’ professional skills, plays a crucial role in improving the independence, quality of life, and wellbeing of elderly individuals with disabilities (Isac et al., 2021). It has been proven that higher levels of spousal empowerment are associated with stronger self-care abilities in disabled elderly (Lehto-Niskala et al., 2022). Therefore, spousal empowerment is not only a key component of family-based care but also an effective means to enhance the SWB of disabled elderly.

Recent evidence further suggests that frailty intersects with disability to exacerbate declines in both physical and mental aspects of QoL (Mei et al., 2025), underscoring the need for holistic interventions targeting psychosocial resources, such as spousal empowerment. Previous studies have separately examined the impact of spousal empowerment on caregivers’ quality of life (Chen et al., 2023) and the relationship between caregiving and subjective wellbeing (SWB) in Non-Asian countries (Fernández Lorca and Lay, 2020; Hajek and König, 2016). However, existing research has yet to thoroughly explore the relationship between the quality of life, SWB, and spousal empowerment of disabled elderly. SWB in individuals with disabilities not only influences their rehabilitation but also correlates with family caregiving burdens. However, prior studies predominantly focused on individual psychological factors, overlooking the role of spousal empowerment as a key familial resource (Funke, 2019; Kawada and Nojima, 2020). Given that spouses represent the most significant source of support, their role cannot be overlooked.

Although the QoL-SWB relationship is well-documented, whether spousal empowerment mediates this association remains unclear, particularly in populations with disabilities. This study aims to: (1) examine the direct effect of QoL on SWB; (2) analyze the mediating role of spousal empowerment; and (3) explore the moderating effect of disability duration. Comprehensively investigating the relationships between these factors and the role of spousal empowerment in the relationship between quality of life and SWB holds substantial theoretical and practical value for developing tailored intervention strategies and elevating the SWB of disabled elderly.

## 2 Method

### 2.1 Participants

A convenience sampling method was employed to select disabled elderly and their spouses residing in a district of Guangzhou, China, from September 2022 to January 2024. The survey was supported by the district Disabled Persons’ Federation, involving home visits coordinated by community committee staff, volunteers from the federation, and the researchers.

Inclusion criteria for disabled elderly included ① Aged ≥ 60 years; ② Barthel Index (BI) ≤ 60 (denoting impaired activities of daily living); ③ No cognitive or communication impairments, with basic cognitive abilities; ④ Informed consent and voluntary participation. Exclusion criteria were ① In the terminal stage of life; ② In an acute phase of illness; ③ Diagnosed with dementia; ④ A history of psychiatric disorders.

For the spouses, inclusion criteria comprised of ① Primary caregiver; ② No cognitive or communication impairments, with basic cognitive abilities; ③ Signed informed consent and voluntary participation in the study. Exclusion criteria included ① Suffering from severe physical illnesses; ② Not living with the disabled elderly; ③ A history of psychiatric disorders.

### 2.2 Sample size

According to statistical guidelines, the recommended sample size is 5 to 10 times the number of variables. Accounting for a 5% invalid return rate, at least 226 samples should be included. In this study, 350 questionnaires were distributed, with 341 returned (response rate: 97.4%). After excluding nine invalid ones, 332 valid questionnaires were obtained, with a response rate of 94.9%. This study was approved by the Ethics Committee of the Fifth Affiliated Hospital, Southern Medical University (Approval No: 2022-HLB-K-001).

### 2.3 Instrument

#### 2.3.1 Questionnaire survey

The questionnaires were designed based on a literature review to collect basic information about disabled elderly and their spouses. The questionnaire for disabled elderly includes gender, age, education level, religious affiliation, residence, number of children, whether they live with children, number of chronic diseases, causes of disability, duration of disability, monthly per capita household income, type of medical payment, and access to medical care. The questionnaire for spouses involves information on gender, age, education level, religious affiliation, number of chronic diseases, daily caregiving hours, total caregiving duration (in months), and whether there are additional co-caregivers.

#### 2.3.2 BI

The BI, developed by Mahoney and Barthel (1965) in 1965, is used to assess activities of daily living (ADL) in elderly individuals. The scale consists of 10 items, covering tasks such as bathing, feeding, and dressing. With a maximum value of 100, the score corresponds to five levels of dependence: 0–20: total dependence; 21–40: substantial assistance required; 41–60: partial independence with moderate assistance needed; 61–99: basic independence with mild impairment; 100: full independence. The BI has demonstrated high reliability and validity and is commonly adopted in evaluating patients with conditions such as stroke, Parkinson’s disease, and Alzheimer’s disease. In this study, Cronbach’s coefficient *α* for the BI was 0.895.

#### 2.3.3 WHOQOL-OLD

The WHOQOL-OLD scale was developed by WHO based on the WHOQOL-BREF to assess the quality of life of individuals aged 60 or above (Power et al., 2005). The scale consists of 6 dimensions: Sensory Abilities, Autonomy, Death and Dying, Past, Present, and Future Activities, Social Participation, and Intimate Relationships, totaling 24 items. A 5-point Likert scale is utilized. A higher score represents a better quality of life. The Chinese version, after translation and cultural adaptation, demonstrates a Cronbach’s coefficient α of 0.892 for the entire scale (Lin and Fang, 2011). In this study, Cronbach’s coefficient α was 0.967 for the WHOQOL-OLD scale and between 0.817 and 0.959 for the dimensions.

#### 2.3.4 MUNSH

The MUNSH scale was developed by Kozma and Stones (1980) in 1980 to assess SWB among elderly individuals. The Chinese version was translated and adapted by Liu and Gong (1999) in 1999. It includes four dimensions: Positive Affect (PA), Negative Affect (NA), Positive Experience (PE), and Negative Experience (NE), totaling 24 items. The total score ranges from 0 to 48. Based on the score, happiness is classified into three levels: low (a score ≤12), moderate (a score between 13 and 35), and high (a score ≥36). For calculation convenience, a constant of 24 is typically incorporated. The overall happiness index is expressed as (PA - NA + PE - NE) + 24. A higher score indicates greater happiness. The scale has demonstrated good reliability and content validity and is extensively used in research on the mental health of elderly individuals (Martín-María et al., 2021). It has a Cronbach’s coefficient *α* of 0.802. In this study, Cronbach’s coefficient *α* for the MUNSH scale was 0.894, with the values of *α* for the dimensions ranging from 0.684 to 0.785.

#### 2.3.5 MCEM

The MCEM scale, developed by Wu and Morikou (2009) under the Chinese cultural context, is used to evaluate the empowerment of caregivers. The scale encompasses nine dimensions: Personal Resources, Caregiver Agency, Caregiving Beliefs, Caregiving Knowledge and Skills, Concerns about Surroundings, Relationship with Care Recipient, Altruistic Care, Perceived Impact of Caregiving, and Expectations of Caregiving Outcomes, totaling 51 items. It employs a 4-point Likert scale. The total score ranges from 51 to 204. A higher score denotes stronger empowerment. The scale has demonstrated high reliability, with a Cronbach’s coefficient α of 0.89. In this study, Cronbach’s coefficient α for the MCEM scale was 0.974, and the values of α for the dimensions were between 0.830 and 0.957.

### 2.4 Quality control

Before the survey, participants were fully briefed on the study’s content and objectives to ensure their informed consent. They were assured of strict confidentiality and data protection. After obtaining consent, the researchers provided standardized instructions on completing the questionnaires and addressed questions from participants. To ensure response accuracy, participants completed questionnaires independently whenever possible. For those requiring assistance due to physical limitations (e.g., visual impairment or motor dysfunction), trained researchers read questions aloud in a neutral tone without interpretation. A standardized training manual was followed to prevent cueing, and all responses were recorded verbatim. Provide training for each researcher before the research begins, we ensured methodological consistency through standardized assistance protocols to minimize bias. The questionnaires were reviewed on-site to ensure all items were answered.

### 2.5 Statistical analysis

Descriptive statistics were used to summarize the demographic characteristics of the sample. Group differences were analyzed using independent t-tests, one-way ANOVA, or non-parametric tests. Pearson correlation analysis was initially conducted to assess the correlations between spousal empowerment, quality of life, and SWB of disabled elderly. Furthermore, multiple linear regression was employed to identify the effect of spousal empowerment on the quality of life and SWB of disabled elderly. Additionally, a mediation model was established using AMOS 29.0 to visually demonstrate the mediating role of spousal empowerment in the relationship between quality of life and SWB. Iterative adjustment with modification indices (MIs) was adopted to optimize the model’s fit and explanatory power. Finally, the robustness of the mediation effect was validated using the Bootstrap method with 5,000 bootstrap samples.

## 3 Result

### 3.1 Demographic characteristics and SWB discrepancy

This study included 332 disabled elderly individuals and their spouses, aged 60 to 94 years, with a mean age of 73.97 ± 6.88. The one-way analysis revealed significant differences in SWB across several demographic variables, including gender, age, education level, residence, number of chronic diseases, duration of disability, monthly per capita household income, type of medical payment, accessibility to medical care, and degree of disability (*p* < 0.05), as listed in Table 1.

**Table 1:** Demographic characteristics and SWB discrepancy among disabled elderly (*n* = 332).

Variables	Categories	Frequency	Proportion (%)	Mean ±SD	t/F	P	LSD/T2 (M)
Gender	Male	183	55.12	25.03 ± 8.95	2.04	0.042	
	Female	149	44.88	23.02 ± 8.93			
Age	60–69 years^a^	96	28.92	24.71 ± 9.49	4.544	0.004	a > c** b > c***
	70–79 years^b^	168	50.60	25.10 ± 9.12			
	80–89 years^c^	60	18.07	20.33 ± 6.61			
	≥90 years^d^	8	2.41	25.38 ± 9.49			
Religious affiliation	Yes	328	98.80	24.11 ± 9.02	−0.307	0.759	
	No	4	1.20	25.50 ± 5.97			
Education level	Illiterate^a^	35	10.54	21.14 ± 7.48	7.468	<0.001	a,b < c,d** a,b < e*** c,d < e*
	Primary school^b^	174	52.41	22.58 ± 8.34			
	Junior high school or vocational school^c^	69	20.78	26.54 ± 9.29			
	Senior high school or technical secondary school^d^	40	12.05	26.58 ± 9.30			
	College or above^e^	14	4.22	32.00 ± 10.05			
Residence	Urban	218	65.66	24.89 ± 9.18	2.144	0.033	
	Rural	114	34.34	22.68 ± 8.44			
Number of chronic diseases	None^a^	37	11.14	28.68 ± 10.90	6.833	0.008	a > c*
	1-2types^b^	77	23.19	24.96 ± 10.56			
	≥3types^c^	218	65.66	23.06 ± 7.71			
Number of children	0	9	2.71	21.11 ± 6.49	2.285	0.112	
	1-2children	113	34.04	25.49 ± 9.90			
	≥3children	210	63.25	23.53 ± 8.48			
Whether living with children	Yes	184	55.42	23.85 ± 8.89	−0.636	0.525	
	No	148	44.58	24.48 ± 9.11			
Duration of disability	<6 months^a^	61	18.37	28.18 ± 10.27	8.987	<0.001	a > c** a > d*** b > d*
	6–12 months^b^	64	19.28	26.03 ± 10.43			
	1–3 years^c^	128	38.55	22.99 ± 8.13			
	>3years^d^	79	23.80	21.30 ± 6.30			
Cause of disability	Disease	317	95.48	24.19 ± 8.93	0.551	0.577	
	Accidental injury	3	0.90	27.00 ± 17.69			
	Others	12	3.61	21.83 ± 8.64			
Monthly per capita household income	≤1000 yuan^a^	41	12.35	18.80 ± 6.54	8.249	<0.001	a < b,d*** a < c** b < d*
	1,001–3000 yuan^b^	150	45.18	23.97 ± 7.98			
	3,001–5000 yuan^c^	81	24.40	24.62 ± 10.72			
	≥5001 yuan^d^	60	18.07	27.52 ± 8.66			
Type of medical payment	Public healthcare^a^	45	13.55	26.98 ± 9.22	4.368	0.016	a,b > c** a,b > d*
	Employee health insurance^b^	72	21.69	26.68 ± 10.02			
	Urban residents’ health insurance^c^	199	59.94	22.79 ± 8.17			
	Five guarantees household^d^	11	3.31	20.27 ± 7.19			
	Others^e^	5	1.51	23.40 ± 13.83			
Accessibility of medical care	Very convenient^a^	182	54.82	25.38 ± 9.32	4.012	0.019	a > b**
	Relatively convenient^b^	108	32.53	22.55 ± 8.36			
	Inconvenient^c^	42	12.65	22.76 ± 8.38			
Degree of disability	Moderate disability^a^	129	40.36	26.64 ± 10.46	8.615	0.001	a > b* a > c**
	Severe disability^b^	69	20.78	22.74 ± 8.25			
	Complete disability^c^	134	38.86	22.43 ± 7.13			

*** indicates *p* < 0.001; ** indicates *p* < 0.01; * indicates *p* < 0.05.

### 3.2 Evaluation results of quality of life, SWB, and spousal empowerment among disabled elderly

The WHOQOL-OLD scores range from 37 to 110, with a mean score of 72.07 ± 19.79, reflecting a moderate level. The SWB scores are between 8 and 45, with a mean value of 24.13 ± 8.98, indicating a level slightly below average. The MCEM scores range from 65 to 190, with a mean score of 140.92 ± 29.13, denoting a moderate level.

### 3.3 Correlation analysis of quality of life, SWB, and spousal empowerment among disabled elderly

Quality of life is positively correlated with the total score of SWB, the PA and PE dimensions, and spousal empowerment (*r* = 0.338, *r* = 0.318, *r* = 0.362, *r* = 0.256, *p* < 0.01). It is negatively correlated with the NA and NE dimensions of SWB (*r* = −0.197, *r* = −0.238, *p* < 0.01). Spousal empowerment is positively linked to the total score of SWB, the PA and PE dimensions (*r* = 0.288, *r* = 0.197, *r* = 0.239, *p* < 0.01) and is negatively related with the NA and NE dimensions of SWB (*r* = −0.266, *r* = −0.252, *p* < 0.01). The results are presented in Table 2.

**Table 2:** Correlation analysis of quality of life, SWB, and spousal empowerment among disabled elderly (*n* = 332).

Variables	1	1.1	1.2	1.3	1.4	1.5	1.6	2	2.1	2.2	2.3	2.4	3	3.1	3.2	3.3	3.4	3.5	3.6	3.7	3.8	3.9
1 WHOQOL-OLD	1																					
1.1 Sensory abilities	0.896**	1																				
1.2 Autonomy	0.930**	0.812**	1																			
1.3 Death and dying	0.726**	0.567**	0.554**	1																		
1.4 Past, present, and future activities	0.910**	0.767**	0.864**	0.550**	1																	
1.5 Social participation	0.913**	0.846**	0.863**	0.562**	0.804**	1																
1.6 Intimate relationships	0.772**	0.556**	0.706**	0.479**	0.766**	0.581**	1															
2 SWB	0.338**	0.283**	0.281**	0.316**	0.268**	0.290**	0.299**	1														
2.1 PA	0.318**	0.277**	0.266**	0.272**	0.253**	0.285**	0.280**	0.865**	1													
2.2 NA	−0.197**	−0.156**	−0.164**	−0.191**	−0.170**	−0.149**	−0.192**	−0.845**	−0.641**	1												
2.3 PE	0.362**	0.317**	0.299**	0.367**	0.262**	0.318**	0.286**	0.754**	0.626**	−0.434**	1											
2.4 NE	−0.238**	−0.185**	−0.197**	−0.216**	−0.200**	−0.205**	−0.230**	−0.849**	−0.625**	0.723**	−0.442**	1										
3 MCEM	0.256**	0.195**	0.217**	0.262**	0.227**	0.210**	0.216**	0.288**	0.197**	−0.266**	0.239**	−0.252**	1									
3.1 Personal resources	0.225**	0.178**	0.187**	0.214**	0.211**	0.189**	0.189**	0.142**	0.092	−0.131*	0.140*	−0.110*	0.826**	1								
3.2 Caregiver agency	0.220**	0.168**	0.192**	0.233**	0.188**	0.183**	0.174**	0.264**	0.181**	−0.266**	0.196**	−0.230**	0.878**	0.660**	1							
3.3 Caregiving beliefs	0.181**	0.118*	0.156**	0.212**	0.179**	0.143**	0.136*	0.274**	0.182**	−0.252**	0.208**	−0.262**	0.828**	0.593**	0.774**	1						
3.4 Caregiving knowledge and skills	0.197**	0.153**	0.159**	0.177**	0.179**	0.198**	0.148**	0.165**	0.119*	−0.166**	0.115*	−0.143**	0.728**	0.726**	0.657**	0.522**	1					
3.5 Concerns about surroundings	0.223**	0.163**	0.205**	0.209**	0.209**	0.148**	0.239**	0.223**	0.164**	−0.196**	0.181**	−0.197**	0.762**	0.576**	0.578**	0.557**	0.475**	1				
3.6 Relationship with care recipient	0.225**	0.178**	0.185**	0.234**	0.176**	0.170**	0.219**	0.300**	0.224**	−0.255**	0.265**	−0.252**	0.865**	0.602**	0.748**	0.692**	0.523**	0.638**	1			
3.7 Altruistic care	0.264**	0.204**	0.229**	0.254**	0.228**	0.232**	0.217**	0.285**	0.180**	−0.267**	0.242**	−0.250**	0.862**	0.625**	0.748**	0.713**	0.570**	0.575**	0.752**	1		
3.8 Perceived impact of caregiving	0.220**	0.184**	0.199**	0.204**	0.189**	0.204**	0.146**	0.274**	0.183**	−0.275**	0.201**	−0.246**	0.868**	0.741**	0.709**	0.650**	0.644**	0.637**	0.720**	0.713**	1	
3.9 Expectations of caregiving outcomes	0.146**	0.095	0.102	0.210**	0.125*	0.099	0.126*	0.247**	0.162**	−0.204**	0.231**	−0.217**	0.816**	0.518**	0.698**	0.711**	0.398**	0.622**	0.755**	0.721**	0.622**	1

** indicates *p* < 0.01; * indicates *p* < 0.05.

### 3.4 Multiple linear regression analysis of factors affecting SWB

With SWB as the dependent variable, the model was built through multiple linear regression analysis. Dummy variables were created for unordered categorical variables to ensure the model’s validity. The results of the regression analysis showed that the model was statistically significant (*F* = 6.446, *p* < 0.001), with no evidence of multicollinearity among the variables (VIF < 10, Tolerance > 0.1). Under the criteria (Entry = 0.05, Removal = 0.10), spousal empowerment had the most significant impact on the SWB of elderly individuals with disabilities, followed by disability duration and education level. These independent variables collectively explained 19.8% of the variance in SWB. The regression equation is simplified as follows: SWB = 0.282 + 0.003 × MCEM - 0.051 × Disability Duration. It demonstrates the impact of key factors on SWB. The information is detailed in Table 3.

**Table 3:** Multiple linear regression analysis of factors affecting the SWB of disabled elderly (*n* = 332).

Independent variable	Unstandardized beta	Standard error	Standardized beta	*t*	*p*
MCEM	0.003	0.001	0.258	4.703	<0.001
Duration of disability	−0.051	0.024	−0.142	−2.15	0.032
Education level	0.053	0.028	0.139	1.939	0.053
WHOQOL-OLD	0.002	0.001	0.122	1.824	0.069
Monthly per capita household income	0.039	0.03	0.097	1.331	0.184
Resistance (with urban areas as the reference)					
Rural	0.061	0.051	0.077	1.187	0.236
Degree of disability	0.025	0.025	0.06	1.011	0.313
Age	−0.024	0.026	−0.047	−0.895	0.372
Accessibility of medical care	−0.024	0.031	−0.045	−0.772	0.441
Type of medical payment (With public medical insurance as the reference)					
Others	0.137	0.171	0.045	0.803	0.423
Urban residents’ health insurance	0.026	0.078	0.034	0.334	0.738
Employee health insurance	0.027	0.066	0.03	0.415	0.678
Five guarantees household	−0.031	0.135	−0.015	−0.229	0.819
Gender (With males as the reference)					
Female	−0.023	0.039	−0.031	−0.589	0.556
Number of chronic diseases	−0.001	0.035	−0.003	−0.039	0.969
*R*^2^ (△R^2^)	0.234 (0.198)				
*F*	6.446				
*p*	<0.001				

### 3.5 The relationship between quality of life, SWB, and spousal empowerment among disabled elderly

#### 3.5.1 Fit of the initial structural equation model

This study constructed a structural equation model based on social support theory. The fit of the initial structural equation model showed: χ^2^/df = 3.816, RMSEA = 0.092, GFI = 0.837, NFI = 0.890, revealed that some fit indices did not meet the ideal standards, necessitating modification.

#### 3.5.2 Model modification

**Figure 1:**
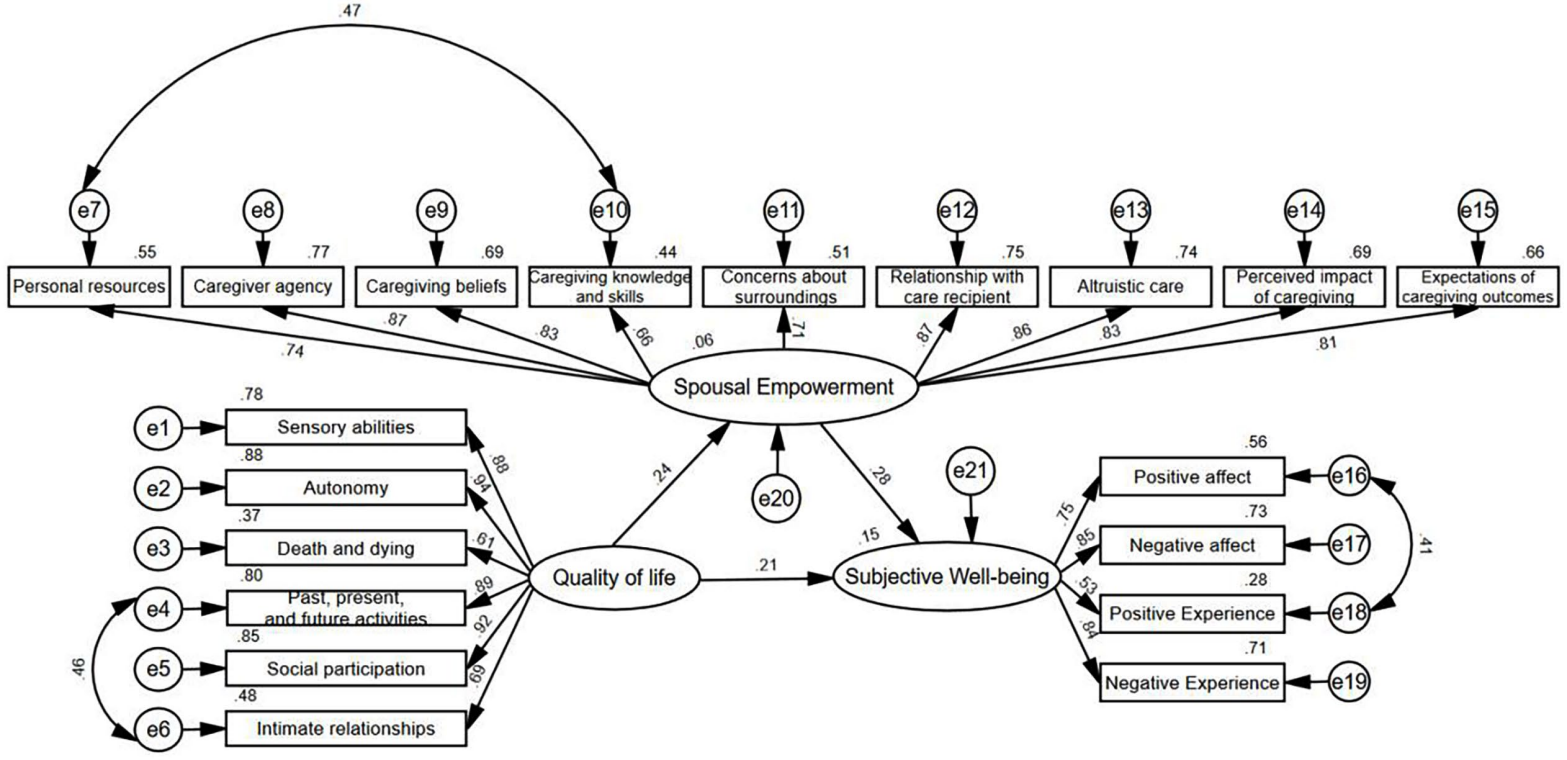
The revised structural equation model (Standardized).

Based on the results of the initial model fitting and MIs, the model was revised three times. The following observed variables were modified: “Personal resources” and “Caregiving knowledge and skills” in the MCEM, “Past, present, and future activities” and “Intimate relationships” in the WHOQOL-OLD, and “PA” and “PE” in the MUNSH. The fit indices showed significant improvement: χ^2^/df < 3; RMSEA < 0.08; NFI, IFI, and CFI all exceeded 0.9; GFI was close to 0.9; PNFI, PCFI, and PGFI were greater than 0.5. The results indicated a good model fit. Further details are provided in Figure 1 and Table 4.

**Table 4:** Fit indices of the revised structural equation model.

	Absolute Fit Index			Incremental Fit Index			Parsimonious Fit Index		
Model	χ2/df	RMSEA	GFI	NFI	IFI	CFI	PNFI	PCFI	PGFI
Criteria (Excellent)	<3	<0.08	>0.9	>0.9	>0.9	>0.9	>0.5	>0.5	>0.5
Model score	2.671	0.071	0.890	0.925	0.951	0.951	0.789	0.812	0.684
Fit result	Excellent	Excellent	Good	Excellent	Excellent	Excellent	Acceptable	Acceptable	Acceptable

Other parameters: n = 332; Chi-square = 389.995; p = 0.000; Degrees of freedom = 146.

#### 3.5.3 Path analysis of the structural equation model

The results showed that the quality of life and spousal empowerment of disabled elderly had significantly positive impacts on SWB. Quality of life directly influenced SWB (*β* = 0.208, *p* < 0.001). It also indirectly affected SWB by promoting spousal empowerment (*β* = 0.239, *p* < 0.001), with an indirect effect of 0.067, resulting in a total effect of 0.275. In addition, the direct effect of spousal empowerment on SWB was particularly significant (*β* = 0.281, *p* < 0.001), making it the most prominent path in the model. Detailed information is provided in Tables 5, 6.

**Table 5:** Path coefficients in the structural equation model.

Effect path		Unstandardized coefficient	Standardized coefficient	S.E.	C.R	*p*
MCEM	← WHOQOL-OLD	0.421	0.239	0.104	4.034	<0.001
SWB	← MCEM	0.131	0.281	0.029	4.535	<0.001
SWB	← WHOQOL-OLD	0.170	0.208	0.050	3.374	<0.001
Intimate relationships	←WHOQOL-OLD	1.000	0.690	—	—	—
Social participation	← WHOQOL-OLD	1.742	0.922	0.114	15.263	<0.001
Past, present, and future activities	← WHOQOL-OLD	1.149	0.893	0.061	18.887	<0.001
Death and dying	← WHOQOL-OLD	1.064	0.611	0.100	10.606	<0.001
Autonomy	← WHOQOL-OLD	1.578	0.940	0.099	15.950	<0.001
Sensory abilities	← WHOQOL-OLD	1.754	0.882	0.119	14.690	<0.001
Personal resources	← MCEM	1.000	0.734	—	—	—
Caregiver agency	← MCEM	0.817	0.878	0.050	16.348	<0.001
Caregiving beliefs	← MCEM	0.675	0.830	0.044	15.319	<0.001
Caregiving knowledge and skills	← MCEM	0.432	0.659	0.027	15.724	<0.001
Concerns about surroundings	← MCEM	0.666	0.708	0.051	12.998	<0.001
Relationship with care recipient	← MCEM	0.855	0.867	0.053	16.022	<0.001
Altruistic care	← MCEM	0.843	0.861	0.053	15.971	<0.001
Perceived impact of caregiving	← MCEM	0.797	0.822	0.045	17.860	<0.001
Expectations of caregiving outcomes	← MCEM	0.860	0.816	0.058	14.913	<0.001
PA	← SWB	1.000	0.749	—	—	—
NA	← SWB	1.216	0.854	0.084	14.473	<0.001
PE	← SWB	0.778	0.529	0.069	11.254	<0.001
NE	← SWB	1.314	0.845	0.092	14.341	<0.001

**Table 6:** Effects of various variables on the SWB of disabled elderly.

Variables	Types	WHOQOL-OLD	MCEM
SWB	Direct effect	0.208	0.281
	Indirect effect	0.067	——
	Total effect	0.275	0.281

#### 3.5.4 Mediation effect of spousal empowerment on the relationship between the quality of life and SWB of disabled elderly

This study used the Bootstrap method with 5,000 resampling iterations to explore the mediation effect of spousal empowerment on the relationship between quality of life and SWB. The results revealed that quality of life had a significant indirect effect (*Z* = 3.056, 95% CI: 0.026–0.098) and a significant direct effect (*Z* = 3.036, 95% CI: 0.065–0.289) on SWB, indicating the partial mediating role of spousal empowerment.

## 4 Discussion

### 4.1 Factors influencing SWB

In this study, the SWB of 332 disabled elderly individuals had an average score of 24.13 ± 8.98, indicating a moderately low level, below the Chinese normative standard (Sun et al., 2016). This discrepancy can be attributed to the characteristics of the study population. The normative standard is based on community residents aged 50 and above, most of whom maintain self-care abilities, while this study focused on disabled elderly aged 60 and above. Physical health is significant for elderly individuals and directly affects their SWB, resulting in a lower total score of SWB in this study. Furthermore, compared with Li′s findings (Li et al., 2022), the SWB scores in this study are also lower, likely due to the higher proportion (61.1%) of severely disabled elderly in this study. It has been confirmed (Huang et al., 2022) that physical status is a decisive factor for SWB, and a worsening of health can lead to a decline in SWB. Impaired physiological functions, loss of self-worth, and decreased social adaptability often induce negative emotions, such as anxiety and depression, further reducing SWB. This study found that gender, age, education level, residence, number of chronic diseases, duration of disability, monthly per capita household income, type of medical payment, accessibility to medical care, and degree of disability all significantly impacted the SWB of disabled elderly. Males showed higher SWB scores than females in this study, which aligns with the findings of Emerson and Llewellyn (2023). As a socially vulnerable group, women often exhibit lower resilience when facing negative events, such as disability, leading to lower SWB levels. Additionally, SWB is negatively correlated with the age of disabled elderly (Huang et al., 2022). It is influenced by factors associated with aging, such as physical decline, cognitive impairment, and chronic diseases. Disabled elderly individuals with higher education levels also report higher SWB scores (Gao et al., 2023). They are more adept at seeking social support, clearly expressing their needs, and possess greater psychological resilience, thus coping with difficulties in a more balanced and rational manner. Residence and accessibility of medical care also affect SWB. Disabled elderly individuals living in urban areas and those with easy access to medical care exhibit higher SWB levels (Zhang et al., 2024). This may be attributed to the superior economic conditions, abundant medical resources, and well-developed social services available in urban areas. Disabled elderly individuals who suffer from multiple chronic diseases tend to have lower SWB levels (Srivastava et al., 2021). The long-term burden and increased risk of disability associated with chronic diseases contribute to negative emotions in these individuals. Disabled elderly individuals with lower monthly per capita household income also report lower SWB (Tsuchiya-Ito et al., 2020). A stable income and favorable economic conditions are foundational to SWB, while the financial strain of treating chronic diseases exacerbates this issue. Regarding medical payment types, disabled elderly covered by public health insurance or employee medical insurance have higher SWB (Cheng et al., 2024), likely due to greater social support and reduced economic pressure. In contrast, those covered by urban medical insurance or those classified as “Five Guarantees” (socially assisted elderly with no children) report lower SWB, owing to lower reimbursement rates and lack of stable economic support. The degree of disability is a central factor influencing SWB (Freedman et al., 2012). The more severe the disability, the greater the difficulties faced in daily life, resulting in lower SWB. Efforts should focus on female disabled elderly and those with an advanced age or lower education levels to enhance their SWB. Additionally, improving medical care conditions in rural areas, strengthening chronic disease management, promoting income for the elderly, improving the medical insurance system, and prioritizing early intervention for elderly with mild to moderate disabilities are essential to prevent further deterioration of their condition.

In order to promote the SWB of disabled elderly and alleviate their negative psychological experiences, a multi-faceted approach is necessary, encompassing social support, family care, and government assistance. Communities should prioritize the management of these individuals, while the government should strengthen public welfare for low-income and uninsured disabled elderly by providing additional economic support and humanitarian care. These efforts will narrow the gap between urban and rural areas.

### 4.2 Correlation analysis of the WHOQOL-OLD, SWB, and MCEM of spouses of disabled elderly

The correlation analysis revealed that quality of life was positively correlated with SWB, as well as the PA and PE dimensions. This suggests that the better the quality of life for disabled elderly is, the higher their SWB, positive emotions, and positive experiences are. Conversely, quality of life was negatively correlated with the NA and NE dimensions of their SWB, indicating that low quality of life reflects high levels of negative emotions and experiences. This finding is consistent with Liu’s study (Liu et al., 2023), highlighting multi-morbidity as a prevalent issue among disabled elderly. Aging and diseases often lead to cognitive dysfunction, impaired vision, and mobility difficulties, which undermine their quality of life. Consequently, many disabled elderly individuals need assistance in their daily activities, such as eating, personal hygiene, and mobility. Prolonged suffering from chronic diseases can foster feelings of inferiority and worthlessness, contributing to complex psychological experiences that significantly reduce their SWB. Both this study and previous research demonstrate that the quality of life of disabled elderly can profoundly impact SWB. Since adverse factors accumulate with the degree of disability, the psychological health of disabled elderly must be prioritized. Additionally, as a vulnerable group, elderly individuals with disabilities are often overlooked in both social and daily life, leading to their stronger need for recognition and support.

The correlation analysis in this study showed that the quality of life of disabled elderly was positively correlated with spousal empowerment. This implies that higher spousal empowerment leads to a better quality of life for the disabled elderly and vice versa. These results align with those of Han (2021). However, Lin et al. (2022) found no significant correlation between spousal empowerment and the quality of life of disabled elderly. This is because spousal empowerment is also influenced by the spouse’s circumstances and the elderly individual’s physical and psychological health. Communities and related institutions should adopt frameworks based on social support theory to provide more resources and support for disabled elderly. Both the individuals and their spouses should maintain an optimistic attitude toward life, actively participate in community health education to learn about treatment and prevention strategies, enhance self-management capabilities, and engage in social interactions. These measures can improve spousal empowerment and enhance the quality of life of disabled elderly, creating a positive cycle of health.

Furthermore, this study found that spousal empowerment was positively correlated with SWB, as well as the PA and PE dimensions, while it was negatively correlated with the NA and NE dimensions of SWB. Upon being incorporated into the multiple linear regression model, spousal empowerment presented the most significant impact on SWB. This demonstrates that spouse empowerment can substantially impact the SWB of elderly individuals with disabilities. Low spousal empowerment can adversely affect the physical and psychological health of disabled elderly, as well as their social adaptation and self-realization. Insufficient spousal empowerment cannot provide effective psychological support or convey health knowledge to these individuals, thus limiting their ability to manage their health. Consequently, they may struggle to face disability. Therefore, improving spouse empowerment is crucial for enhancing the SWB of disabled elderly.

### 4.3 The mediating role of *spousal empowerment* in the relationship between the quality of life and SWB of disabled elderly

The results disclosed that spousal empowerment played a mediating role in the relationship between the quality of life and SWB of disabled elderly. Specifically, quality of life not only directly impacted SWB but also exerted an indirect effect on SWB through spousal empowerment. This finding aligns with previous studies, which reported that spousal empowerment was positively linked to the SWB of disabled elderly (Liu et al., 2020; Lin et al., 2022), underscoring the critical role of spouses in caregiving for these individuals.

The decline in physical functions often severely degrades the quality of life of disabled elderly, which is directly correlated with their SWB. Since spouses are the primary caregivers and emotional supporters of disabled elderly, spouse empowerment significantly influences the transformation of these individuals’ quality of life into SWB. Our findings resonate with Iždonaitė-Medžiūnienė et al. (2024), who demonstrated that frailty and cognitive concerns synergistically reduce QoL in older adults, suggesting that spouse-mediated support may buffer these effects through enhanced social engagement or practical assistance. Through effective communication, involvement in rational decision-making, and sensitive responses to the needs of disabled elderly, spouses not only improve their partners’ living conditions but also enhance their psychological resilience and life satisfaction. In turn, this indirectly boosts the SWB of these individuals.

### 4.4 Theoretical contributions and practical implications

This study advances empowerment theory by empirically validating its role in spousal caregivers of disabled elderly, with empowerment partially mediating (*β* = 0.067, 95% CI [0.026, 0.098]) the QOL-SWB relationship—a pathway previously underexplored in gerontological research. Social support theory emphasizes the direct impact of external support (such as emotional and instrumental support) on individual wellbeing. Our findings extend social support theory by highlighting empowerment as a psychological pathway through which spousal support translates into enhanced SWB, shifting the focus from passive receipt of support to active capability-building. Empowerment theory typically focuses on individuals or groups enhancing their sense of control through resource acquisition, but is less validated in the context of elderly care. This study refines empowerment theory by demonstrating its protective role in spousal caregivers of disabled elderly, particularly in mitigating the cumulative stress of long-term disability. The partial mediation model challenges uni-dimensional QOL-SWB frameworks, advocating for integrated models that incorporate relational empowerment processes within families.

Policymakers should consider spouse-specific subsidies (e.g., respite care vouchers) within national caregiver policies, while practitioners could implement gender-sensitive training to address role strain. Community-based peer networks and digital tools (e.g., caregiver apps) may further enhance support accessibility. The following specific suggestions are proposed for policy makers and practitioners to enhance the practical guidance significance of research:

For Policymakers: (1) Integrate spousal empowerment into national care support programs: in existing nursing subsidies or welfare policies, special support for spouses (such as psychological counseling, economic subsidies, and respite services) should be added. Promote legislation to protect the rights of spousal caregivers, such as flexible working hours and paid care leave.(2) Develop gender sensitive policies: Due to gender differences in nursing responsibilities (such as women being more likely to take on nursing roles), policies should provide targeted support (such as skill training for male caregivers and economic empowerment for female caregivers).(3) Promote cross departmental collaboration: Establish a comprehensive support network for spouse caregivers in collaboration with health, social security, community services, and other departments.

For Practitioners: (1) Tailored empowerment interventions: Design skill training for spouse caregivers (such as chronic disease care skills, stress management) and include them in “peer support groups” to enhance social connections. Provide personalized psychological counseling to help spouses cope with role conflicts, such as the pressure of balancing work and care. (2) Community based support models: Conduct regular health check ups and nursing skills workshops through community centers, and establish a “caregiver professional institution” referral mechanism. (3) Utilizing technological means: Develop mobile applications or online platforms to provide nursing knowledge base, remote medical consultation, and emergency contact services.

### 4.5 Limitations and future research directions

This study only employed a cross-sectional questionnaire survey method, lacking qualitative research. In future, longitudinal studies should be conducted to explore the relationships and mechanisms between variables, as well as incorporating qualitative analysis. Moreover, the sample size was relatively small. In order to address this issue, multi-center exploration and intervention studies should be conducted. We acknowledge the exclusion criterion (such as, patients and spouses with psychiatric disorders excluded) may limit generalizability but is critical for internal validity. A future study targeting psychiatric comorbidities is planned.

Future research can explore: (1) Whether other family factors (such as child support) interact with spouse empowerment; (2) Develop a family empowerment intervention plan based on this model and evaluate its effectiveness; (3) Cross cultural comparison to examine the impact of spousal role differences on the path model; (4) The dynamic mediation effect of spouse empowerment can be verified through longitudinal design; (5) Given evidence that subjective wellbeing determinants may vary by gender (e.g., Goda et al., 2025), future studies should explore whether the mediating role of spousal empowerment differs between male and female caregivers.

## 5 Conclusion

The quality of life and SWB of disabled elderly are at moderate to low levels, while spousal empowerment is at a moderate level, all of which need further improvement. Factors such as gender, age, educational level, residence, the number of chronic diseases, duration of disability, monthly per capita household income, type of medical payment, accessibility to medical care, and the degree of disability significantly influence SWB.

Both quality of life and spousal empowerment positively impact the SWB of disabled elderly. Additionally, spousal empowerment partially mediates the relationship between quality of life and SWB. Therefore, comprehensive strategies and personalized interventions focusing on spousal empowerment can effectively improve the SWB of disabled elderly.

## Data Availability

The original contributions presented in the study are included in the article/Supplementary material, further inquiries can be directed to the corresponding author/s.
